# Editorial: Nutricines and derivatives of nutrients in animal health and disease prevention

**DOI:** 10.3389/fvets.2024.1391563

**Published:** 2024-03-12

**Authors:** Tao Wang, Hong-Gu Lee

**Affiliations:** ^1^Key Laboratory of Animal Nutrition and Feed Science of Jilin Province, Key Laboratory of Animal Production Product Quality and Security Ministry of Education, JLAU-Borui Dairy Science and Technology R&D Center, College of Animal Science and Technology, Jilin Agricultural University, Changchun, China; ^2^Laboratory of Animal Nutrition, Physiology and Proteomics, Department of Animal Science and Technology, Konkuk University, Seoul, Republic of Korea

**Keywords:** animal production, bioactive potential, nutrition-based health, self-defense system, underlying mechanisms

Good animal health is of great importance for the efficient production of animal-derived foods at a low cost. The demand for solutions for animal health maintenance and disease prevention is a major global challenge in animal production, food safety, and public health (1). The animal body has a complete self-defense system, including oxidation and anti-oxidation balance, immune activation and suppression balance, pro- and anti-inflammatory balance, which can help the host against various factors that endanger normal life activities (2). Nowadays, the strategy of nutrition-based health has become an increasingly important solution for animal health maintenance and disease prevention (3). Nutrients (carbohydrates, fats, proteins, minerals, vitamins, et al.) and nutricines (carotenoids, enzymes, fatty acids, flavors, oligosaccharides, organic acids, phospholipids, polyphenols, et al.) are two major categories of components in feeds (4, 5).

This Research Topic attracts considerable studies dealing with the underlying mechanisms of nutricines and derivatives of nutrients in the animal self-defense system. And researches on the latest information about novel techniques and theories to identify the bioactive activity of nutricines and derivatives of nutrients are also received. After peer review, a collection of 14 of those studies are published, 11 are original research and three are review.

Interestingly, three articles addressed the potential benefits of yeast (culture) in animal health and disease prevention. Maturana et al., provided the reader with a comprehensive review on the effects of yeast and its derivatives in pets and the possible mechanisms that confer their functional properties. Aschalew et al. reported that yeast culture and oxalic acid have a great potential to buffer and create a conducive rumen environment and improve rumen fermentation efficiency and hemicellulose digestion. Yin et al. proved that inositol, a potentially potent metabolite in yeast culture, can improve rumen function, affect rumen microorganisms and rumen and blood metabolites and may reduce inflammation, improving animal health.

Moreover, two articles reported the roles of polyphenol-rich botanical stuffs in animal nutrition. In the review of Ferlisi et al., the authors gave an update on the use of olive co-products and their phenolic extracts in monogastric animal (swine, poultry and rabbit) diets and suggested that these stuffs may improve animal health, productive performances and meat quality characteristics, reduce the adverse effect of lipid peroxidation and improve the antioxidant status. Wang J. et al. found that dark tea can mitigate oxidative stress-induced damage by promoting the clearance of free radicals and suggested that dark tea is worth further exploration as a potential dietary supplement for the maintenance of animal health and the prevention of related diseases.

In addition, the bioactive potential of two different molecules in the animals' performance were conducted. Wang C. et al. concluded that β-hydroxybutyrate administration might alleviate the liver injury and inflammation, and improve hepatic energy metabolism by regulating glucose and lipid metabolism, thereby improving the growth performance of postnatal growth retardation piglets. Li et al. presented that supplemental β-alanine can improve the antioxidant status of speed-racing Yili horses reduce post-exercise injuries and bolster their post-race recovery ability.

Overall, this Research Topic contributed to improving the current knowledge of nutricines and derivatives of nutrients in animal health and disease prevention, providing significant contribution to this research area.

## Author contributions

TW: Writing – original draft, Writing – review & editing. H-GL: Writing – review & editing.
